# Genomic Approaches to Study Alzheimer’s Disease Immunity

**DOI:** 10.1002/alz.087774

**Published:** 2025-01-03

**Authors:** David Gate

**Affiliations:** ^1^ Northwestern University, Chicago, IL USA

## Abstract

**Background:**

Much attention has been paid to the role of the perenchymal brain immune response in Alzheimer’s disease (AD). Yet, the peripheral immune system in AD has not been thoroughly studied with modern sequencing methods.

**Method:**

Here, we used a combination of single‐cell sequencing strategies, including assay for transposase‐accessible chromatin and RNA sequencing, to investigate the epigenetic and transcriptional alterations to the AD peripheral immune system.

**Result:**

We reveal a striking amount of open chromatin in peripheral immune cells in AD. In CD8 T cells, we uncover a cis‐regulatory DNA element co‐accessible with the CXC motif chemokine receptor 3 gene promoter. In monocytes, we identify a novel RELA transcription factor binding site adjacent to an open chromatin region in the nuclear factor kappa B subunit 2 gene. We also demonstrate apolipoprotein E genotype‐dependent epigenetic changes that correspond to altered cytokine gene expression in monocytes. Surprisingly, we also identify differentially accessible chromatin regions in genes associated with sporadic AD. Finally, we provide an online data portal resource to explore gene expression in the AD immune system.

**Conclusion:**

Our findings provide novel insights into the complex relationship between epigenetics and genetic risk factors in the AD peripheral immune system.

